# A review of mechanisms of disease across *PIK3CA*-related disorders with vascular manifestations

**DOI:** 10.1186/s13023-021-01929-8

**Published:** 2021-07-08

**Authors:** Guillaume Canaud, Adrienne M. Hammill, Denise Adams, Miikka Vikkula, Kim M. Keppler-Noreuil

**Affiliations:** 1 Overgrowth Syndrome and Vascular Anomalies Unit, Hôpital Necker Enfants Malades, INSERM U1151, Assistance Publique-Hôpitaux de Paris, Université de Paris, 149 rue de Sèvres, 75105 Paris, France; 2 Division of Hematology, Cancer and Blood Diseases Institute, Cincinnati Children’s Hospital Medical Center and Department of Pediatrics, University of Cincinnati College of Medicine, Cincinnati, OH USA; 3 Division of Oncology, Comprehensive Vascular Anomalies Program, Children’s Hospital of Philadelphia, Perelman School of Medicine and the University of Pennsylvania, Philadelphia, PA USA; 4 Human Molecular Genetics, de Duve Institute, University of Louvain, Brussels, Belgium; 5 Center for Vascular Anomalies, Division of Plastic Surgery, Cliniques Universitaires Saint Luc, University of Louvain, Brussels, Belgium; 6 VASCERN VASCA European Reference Centre, Bichat-Claude Bernard Hospital, Paris, France; 7 Walloon Excellence in Lifesciences and Biotechnology (WELBIO), University of Louvain, Brussels, Belgium; 8 Division of Genetics and Metabolism, Department of Pediatrics, University of Wisconsin School of Medicine and Public Health, Madison, WI USA

**Keywords:** *PIK3CA*, PI3K, PROS, Vascular malformation, Sirolimus, Alpelisib, Miransertib

## Abstract

**Background:**

*PIK3CA*-related disorders include vascular malformations and overgrowth of various tissues that are caused by postzygotic, somatic variants in the gene encoding phosphatidylinositol-3-kinase (PI3K) catalytic subunit alpha. These mutations result in activation of the PI3K/AKT/mTOR signaling pathway. The goals of this review are to provide education on the underlying mechanism of disease for this group of rare conditions and to summarize recent advancements in the understanding of, as well as current and emerging treatment options for *PIK3CA*-related disorders.

**Main body:**

*PIK3CA*-related disorders include *PIK3CA*-related overgrowth spectrum (PROS), *PIK3CA*-related vascular malformations, and *PIK3CA*-related nonvascular lesions. Somatic activating mutations (predominantly in hotspots in the helical and kinase domains of *PIK3CA,* but also in other domains), lead to hyperactivation of the PI3K signaling pathway, which results in abnormal tissue growth. Diagnosis is complicated by the variability and overlap in phenotypes associated with *PIK3CA*-related disorders and should be performed by clinicians with the required expertise along with coordinated care from a multidisciplinary team. Although tissue mosaicism presents challenges for confirmation of *PIK3CA* mutations, next-generation sequencing and tissue selection have improved detection. Clinical improvement, radiological response, and patient-reported outcomes are typically used to assess treatment response in clinical studies of patients with *PIK3CA*-related disorders, but objective assessment of treatment response is difficult using imaging (due to the heterogeneous nature of these disorders, superimposed upon patient growth and development). Despite their limitations, patient-reported outcome tools may be best suited to gauge patient improvement. New therapeutic options are needed to provide an alternative or supplement to standard approaches such as surgery and sclerotherapy. Currently, there are no systemic agents that have regulatory approval for these disorders, but the mTOR inhibitor sirolimus has been used for several years in clinical trials and off label to address symptoms. There are also other agents under investigation for *PIK3CA*-related disorders that act as inhibitors to target different components of the PI3K signaling pathway including AKT (miransertib) and PI3K alpha (alpelisib).

**Conclusion:**

Management of patients with *PIK3CA*-related disorders requires a multidisciplinary approach. Further results from ongoing clinical studies of agents targeting the PI3K pathway are highly anticipated.

**Supplementary Information:**

The online version contains supplementary material available at 10.1186/s13023-021-01929-8.

## Introduction

*PIK3CA*-related disorders (including *PIK3CA*-related overgrowth spectrum, or PROS) are caused by postzygotic, somatic variants in the gene that encodes phosphatidylinositol-3-kinase (PI3K) catalytic subunit alpha (p110α) [1]. These variants are associated with hyperactivation of the PI3K signaling pathway, which includes multiple downstream effectors such as AKT and mTOR, resulting in vascular malformations and abnormal growth of various tissues [1, 2].

*PIK3CA*-related disorders that fall under the PROS umbrella term are characterized by clinical diagnostic criteria (initially defined by an NIH workshop that convened in 2013): Presence of a somatic *PIK3CA* pathogenic variant (however, tissue mosaicism can be a barrier to mutation confirmation), congenital or early childhood onset, sporadic occurrence, and a spectrum of two or more clinical features [3]. While each PROS disorder is distinct, they often share overlapping clinical features [3]. However, many patients with *PIK3CA*-related vascular malformations or other *PIK3CA*-related lesions do not present with tissue overgrowth [4]. Due to the limitations of the term “PROS,” we propose “*PIK3CA*-related disorders” as more appropriate to describe non-cancerous clinical characteristics/phenotypes and disorders that result from postzygotic, somatic mutations in *PIK3CA*. In addition, this label agrees with recent guidelines for naming genetic disorders [5]. PROS would then be considered a subcategory of *PIK3CA*-related disorders along with *PIK3CA*-related vascular malformations and *PIK3CA*-related nonvascular lesions.

The goals of this review are to provide education on the underlying mechanism of disease for this group of rare disorders, to summarize recent advancements in the understanding of *PIK3CA*-related disorders, and to discuss current and emerging treatment options. A patient-focused, plain language summary of this review can be found in the supplementary information (Additional file 1).

## Methods

In December 2020 and January 2021, literature searches were conducted using PubMed and Google Scholar with search terms including “*PIK3CA*-related overgrowth spectrum,” “PROS,” “*PIK3CA* somatic mutations,” “*PIK3CA* signaling,” “alpelisib,” “miransertib,” and “sirolimus.” ClinicalTrials.gov was searched for relevant trials of alpelisib, miransertib, and sirolimus. Additional references and clinical trials were identified through review of retrieved articles and author knowledge and expertise. This review represents the authors’ perspective based on selected literature. This approach had certain limitations, including a lack of comprehensive, prospectively defined criteria to identify, select, and evaluate references. Hence, the search results used as the basis for the review may not be a complete, objective representation of all the published literature related to the topic.

## Etiology/mechanism of disease for *PIK3CA*-related disorders

**Fig. 1:**
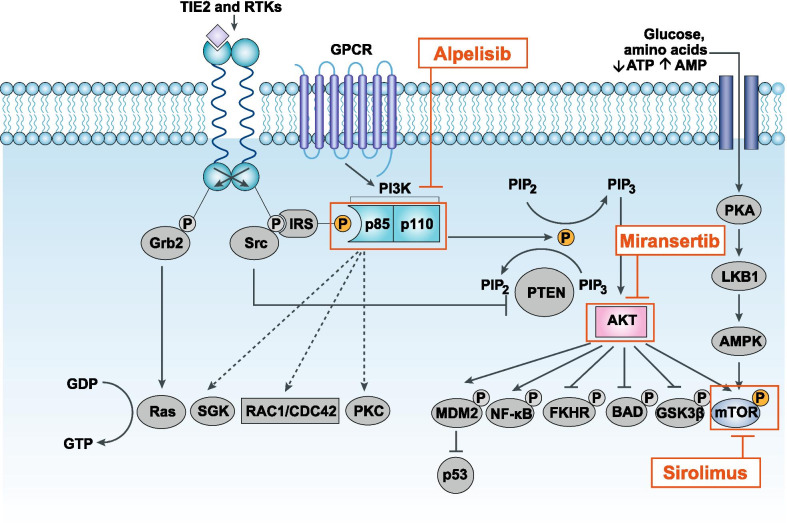
The PI3K signaling pathway and inhibitors under investigation. Because *PIK3CA* mutations underlie the pathogenesis of *PIK3CA*-related disorders, there are multiple strategies for targeting the PI3K pathway under investigation [6, 8]. AKT: protein kinase B; AMP: adenosine monophosphate; AMPK: AMP-activated protein kinase; ATP: adenosine triphosphate; BAD: Bcl-2-associated death promoter; CDC42: Cell division control protein 42 homolog; ERK: extracellular signal regulated kinase; FKHR: forkhead; GDP: guanosine diphosphate; GPCR, G protein-coupled receptor; Grb2: growth factor receptor-bound protein 2; GSK3: glycogen synthase kinase 3; GTP: guanosine-5'-triphosphate; IRS: insulin receptor substrate; LKB1: liver kinase B1; MAPK: mitogen-activated protein kinase; MDM2: mouse double minute 2; mTOR: mammalian target of rapamycin; NF-κB: nuclear factor kappa B; P: phosphate; PI3K: phosphatidylinositol-3-kinase; *PIK3CA*: phosphatidylinositol-4,5-bisphosphate 3-kinase catalytic subunit alpha; PIP_2_: phosphatidylinositol-4,5-bisphosphate; PIP_3_: phosphatidylinositol-3,4,5-triphosphate; PKA: protein kinases A; PKC: protein kinase C; PROS: *PIK3CA*-related overgrowth spectrum; PTEN: phosphatase and tensin homolog; RAC1: Ras-related C3 botulinum toxin substrate 1; Ras: rat sarcoma; RTK, receptor tyrosine kinase; SGK: serum- and glucocorticoid-inducible kinase; Src: rous sarcoma; TIE2, angiopoietin-1 receptor. Adapted with permission from Hennessy 2005 [7]

The PI3K signaling pathway plays a role in many cellular processes, including proliferation, angiogenesis, survival, and metabolism (Fig. 1 [6–8]) [9, 10]. In human cells, three main classes of PI3K are categorized based on their structure and substrate preference [11–13]. Class II PI3Ks and class III PI3Ks contain 3 enzymes (PI3K-C2α, β, γ) and one enzyme (hVPS34), respectively [11]. In addition, a group of serine/threonine kinases including mTOR is sometimes referred to as class IV PI3Ks [13]. Class I PI3Ks are activated downstream of growth factor receptors such as PDGF receptor (PDGFR), epidermal growth factor receptor (EGFR), insulin-like growth factor receptor (IGFR), and insulin receptor (INSR) [11]. The TIE2 tyrosine kinase receptor, which is specific to endothelial cells, also activates PI3K upon angiopoietin-1 binding [4, 14, 15]. Somatic mutations in *TIE2/TEK* have been implicated in vascular malformations [16, 17].

Class I PI3Ks are composed of a catalytic subunit (p110) and a regulatory subunit [11]. The class I p110α isoform, which is encoded by *PIK3CA*, is one of the four isoforms of the p110 catalytic subunit found in mammals (p110α, β, γ, and δ) and is the focus of this review [9, 11]. Class I PI3Ks catalyze the phosphorylation of phosphatidylinositol-4,5-bisphosphate (PIP_2_) to produce phosphatidylinositol-3,4,5-trisphosphate (PIP_3_), which recruits effector proteins such as the AKT subfamily of AGC serine/threonine kinases [9, 11]. Notably, *TIE2* mutations have also been shown to activate AKT [4, 14, 15]. AKT indirectly activates the mTOR serine/threonine kinase, which is found in the cellular complexes mTORC1 and mTORC2 [9]. The tumor suppressor protein phosphatase and tensin homolog (PTEN) suppresses PI3K signaling by dephosphorylating PIP_3_ [9]. Components of the PI3K/AKT/mTOR pathway interact with other signaling pathways, including RAS/RAF/MEK/MAPK [9].

The *PIK3CA* gene encodes the α isoform of the p110 catalytic subunit of PI3K (PI3Kα) and is ubiquitously expressed [9]. The somatic activating mutations in *PIK3CA*-related disorders are also frequently observed in numerous cancers including hotspot mutations in the helical and kinase domains (E542K and E545K in the helical domain; H1047L and H1047R in the kinase domain) [3, 18]. These mutations result in hyperactivation of the PI3K signaling pathway, which leads to abnormal growth of tissues, including epithelial and mesenchymal cells [9, 19]. Preclinical studies have shown that these mutations result in AKT activation, changes in cell morphology, and downregulation of angiogenic factors [4, 20]. The resulting overgrowth syndromes often include slow-flow vascular malformations such as lymphatic and venous malformations [19–21]. Studies of venous malformations (VM) show that activation of the TIE2/PI3K pathway promotes growth of human umbilical vein endothelial cells in xenograft models, aberrant expression of genes that regulate vascular development (including platelet-derived growth factor, β polypeptide), and disruption of the endothelial cell monolayer [14, 15, 19, 22]. Relative to other *PIK3CA*-related disorders, the variants observed in patients with megalencephaly-capillary malformation (MCAP/M-CM) are often seen in non-hotspot loci on the *PIK3CA* gene and are associated with less-activating mutations [23, 24].

The postzygotic, sporadic mutations that occur in *PIK3CA*-related disorders produce genetically distinct cell lineages found in a mosaic pattern based on the developmental stage in which they occur [9, 12, 24–26]. Overall, the variant, timing, and location of these mutations likely contribute to the basis for the wide-ranging manifestations observed in *PIK3CA*-related disorders [20]. Due to the heterogeneity of these disorders and the wide variety of affected tissues, patients may have different responses to therapeutic intervention; however, prospective, controlled studies are needed.

## Classification of *PIK3CA*-related disorders and relationship to vascular anomaly classifications

**Fig. 2:**
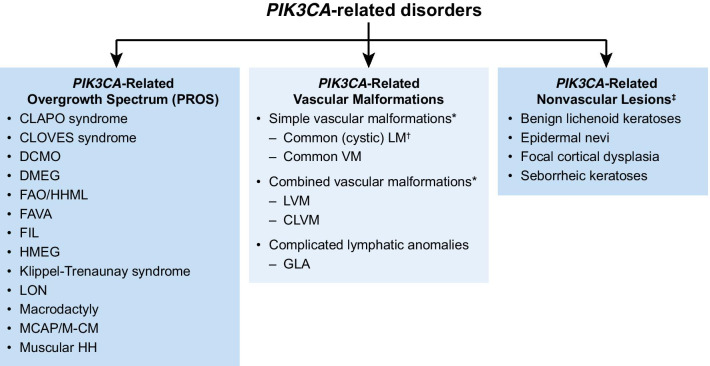
*PIK3CA*-related disorders. The category of *PIK3CA*-related disorders can be divided into 3 subcategories: PROS, *PIK3CA*-related vascular malformations, and *PIK3CA*-related nonvascular lesions. CLAPO: capillary malformation of the lower lip, lymphatic malformation of the face and neck, asymmetry and partial/generalized overgrowth; CLOVES: congenital lipomatous overgrowth, vascular malformations, epidermal nevi, scoliosis/skeletal and spinal; CLVM: combined capillary-lymphatic-venous malformation; DCMO: diffuse capillary malformation with overgrowth; DMEG: dysplastic megalencephaly; FAO/HHML: fibroadipose hyperplasia or overgrowth/hemihyperplasia-multiple lipomatosis; FAVA: fibroadipose vascular anomaly; FIL: fibroadipose or facial infiltrating lipomatosis; GLA, generalized lymphatic anomaly; HH: hemihyperplasia; HMEG: hemimegalencephaly; LM, lymphatic malformation; LON: lipomatosis of nerve; LVM: combined lymphatic-venous malformation; MCAP: megalencephaly-capillary malformation; *PIK3CA*: phosphatidylinositol-4,5-bisphosphate 3-kinase catalytic subunit alpha; VM, venous malformation. *Malformations that are composed of only one type of vessel are classified as “simple” (with the exception of arteriovenous malformation). If a lesion contains ≥ 2 types of vascular malformations, it is classified as “combined.” Vascular malformations are also classified according to major named vessels or by association with other anomalies (e.g., PROS) Wassef 2015 [33]. †Some common (cystic) LM are classified as PROS when they are associated with overgrowth ISSVA 2018 [2]. ‡Can occur as isolated lesions or with other clinical features. Supporting Sources: *PIK3CA*-related overgrowth spectrum: Keppler-Noreuil 2015 [3]; ISSVA 2018 [2]; Goss 2020 [28]; Hughes 2020 [29]; Mahan 2014 [30]; Rios 2013 [31]; Rodriguez-Laguna 2019 [32]; *PIK3CA*-related vascular malformations: ISSVA 2018 [2]; Rodriguez-Laguna 2019 [32]; *PIK3CA*-related nonvascular lesions: Keppler-Noreuil 2015 [3]; Combined vascular malformations: Wassef 2015 [33]; Brandigi 2018 [27]

Currently identified *PIK3CA*-related disorders are presented in Fig. 2 [2, 3, 27–33]. According to the classification from the International Society for the Study of Vascular Anomalies (ISSVA), PROS disorders include the following: Congenital lipomatous overgrowth, vascular malformations, epidermal nevi, scoliosis/skeletal and spinal (CLOVES) syndrome; dysplastic megalencephaly (DMEG); fibroadipose hyperplasia or overgrowth (FAO); fibroadipose infiltrating lipomatosis/facial infiltrative lipomatosis (FIL); hemihyperplasia multiple lipomatosis (HHML); Klippel-Trenaunay syndrome (KTS); macrodactyly and MCAP/M-CM [2]. The ISSVA classification also lists other vascular anomalies associated with *PIK3CA* variants: CLAPO syndrome (lower lip capillary malformation + face and neck lymphatic malformation + asymmetry and partial/generalized overgrowth); common (cystic) lymphatic malformation (LM); common VM; and fibroadipose vascular anomaly (FAVA) [2]. Emerging evidence suggests that *PIK3CA* mutations can be found in combined lymphatic-venous malformations (LVM) and combined capillary-lymphatic-venous malformations (CLVM) – malformations that are also found in patients with KTS [24, 27, 33]. In addition, the following syndromes and/or isolated phenotypes are classified as *PIK3CA*-related according to the 2013 NIH workshop: hemimegalencephaly (HMEG), muscular hemihyperplasia (HH), seborrheic keratoses (SK), epidermal nevi (EN), and benign lichenoid keratoses (BLK). Since the latter three phenotypes can occur as isolated features we have categorized these as *PIK3CA*-related nonvascular lesions [3]. *PIK3CA* mutations have also been implicated in some cases of diffuse capillary malformation with overgrowth (DCMO), lipomatosis of nerve (LON), and some complicated lymphatic anomalies (CLAs) including generalized lymphatic anomaly (GLA) [28–30, 32].

## Diagnosis and complications associated with *PIK3CA*-related disorders

Clinical diagnosis is complicated by the variability and overlapping phenotypes of *PIK3CA*-related disorders [3, 34–36]. Diagnosis should be performed by clinicians with expertise followed by coordinated care from a multidisciplinary team, which includes surgeons, radiologists, geneticists, dermatologists, pathologists, and hematologists/oncologists, the latter of whom are critical for the emerging medical management and coordination of the associated long-term follow-up [6, 35–37].

Key criteria for diagnosis of PROS disorders have been outlined by Keppler-Noreuil et al. [3]. Simple vascular malformations, including common (cystic) LMs and common VMs, are composed of only one type of vessel, whereas combined vascular malformations, including CLVMs and LVMs, are composed of combinations of vessels. Both may be characterized by clinical and radiological features outlined by ISSVA [33]. A distinct, multidisciplinary set of guidelines for evaluation of CLAs was recently published [38]. The main imaging modalities used to diagnose subcutaneous vascular malformations are Doppler ultrasound and magnetic resonance imaging [39]. For *PIK3CA*-related nonvascular lesions, specialists should be consulted depending on location of the lesion.

Clinicians should take a careful patient history, including a family history to distinguish between sporadically occurring and inherited disorders [25, 36, 37]. Confirmation of *PIK3CA* mutation is highly recommended for diagnosis of *PIK3CA*-related disorders; however, a negative test result does not preclude diagnosis [3]. Due to the mosaic nature of *PIK3CA*-related disorders and challenges with tissue biopsy collection, it may not always be possible to confirm the presence of the *PIK3CA* mutation [3]. Given the low level of mosaicism in some patients, next-generation sequencing or specific droplet digital PCR (ddPCR) for hot spot mutations may be the optimal techniques for identifying mutations [4, 24, 34, 40–42].

It is important to note that the majority of patients with *PIK3CA*-related disorders experience a progressive disease course, with clinical manifestations typically observed within the patient’s first year of life, with additional manifestations becoming apparent later [3, 9]. This could pose a challenge to diagnosis and ongoing care of patients with an early diagnosis of vascular malformation, who go on to experience overgrowth or other manifestations in different tissues. When considering the nomenclature for these disorders, care should be taken to consider this possibility.

In the differential diagnosis of *PIK3CA*-related disorders, it is important to consider other syndromes with overlapping characteristics but different genetic causes, such as Proteus syndrome and PTEN Hamartoma Tumor Syndrome [3]. Proteus syndrome is associated with a somatic activating *AKT1* mutation and has distinct clinical findings [43]. PTEN Hamartoma Tumor Syndrome is associated with vascular anomalies in more than 50% of patients as well as fat overgrowth, which can result in misdiagnosis as PROS disorders, such as CLOVES or FAVA [44]. In addition, there are overlapping clinical phenotypes caused by somatic mutations in other related gene pathways, such as in the mosaic RASopathies [45–49].

Complications that are commonly associated with *PIK3CA*-related disorders, which may or may not be related to vascular malformations, are detailed in Table 1 [1, 3, 9, 42, 50]. Based on the patient’s presentation, clinical imaging evaluations should be performed as needed [3, 51].

**Table 1:** Complications associated with *PIK3CA*-related disorders [1, 3, 9, 42, 50, 52]

Severe functional impairment
Pain
Vascular complications
Coagulopathy or changes in coagulation Chronic consumptive coagulopathy Localized intravascular coagulopathy
Hemorrhage
Thromboembolism
Infections
Neurologic complications
Ventriculomegaly or hydrocephalus
Cerebellar tonsillar ectopia
Brainstem compression
Seizures
Developmental delay Hypotonia
Risk of malignant tumors
Wilms’ tumor
Leukemia
Meningiomas
Dermatologic, genitourinary, gastrointestinal, or endocrine/metabolism involvement

## Challenges of assessing treatment response

Clinical improvement, radiological response, and patient-reported outcomes are typically used to assess treatment response in clinical studies of patients with *PIK3CA*-related disorders [22, 36, 53–57]. However assessment of individual patients over time is complicated by the variable natural histories of these disorders and incomplete resolution of symptoms [53]. Accurate and thorough assessment of patient history is also critical to rule out vascular malformations caused by inherited mutations (e.g., hereditary hemorrhagic telangiectasia, glomuvenous malformation, and CM-AVM) or other congenital conditions with similar clinical features or symptoms [36, 57]. Even when properly characterized, these conditions are both rare and highly heterogeneous, which poses challenges to recruitment and completion of clinical studies [53].

Clinical diagnosis of venous malformations can be a significant challenge. D-dimer is frequently elevated in patients with venous malformations and has emerged as a biomarker to aid differential diagnosis [51, 58]. Elevated D-dimer can differentiate KTS from Parkes Weber syndrome as well as multifocal venous lesions versus glomuvenous malformations [58]. Patients with venous malformations are at risk for coagulopathy, and those with elevated levels of D-dimer and low levels of fibrinogen are at particular risk for hemorrhage [52]. Levels of both D-dimer and fibrinogen have been shown to improve in patients with *TIE2*–mutated and *PIK3CA-*mutated venous malformations treated with sirolimus [22, 54], suggesting that these markers could be used to monitor response to treatment.

When assessing response with imaging techniques, growth over time and interpatient provides challenges to objective assessments [1, 54]. Notably, even when clinical improvement and successful treatment of symptoms is observed, assessments may continue to appear abnormal [54, 59]. As a result, patient-reported outcome assessments such as quality-of-life questionnaires and pain assessment by visual analog scale may be the best tools to measure efficacy, but these may be confounded effects due to many patients experiencing symptoms since birth [54]. There is an unmet need for validated patient-reported outcome tools that are specifically designed to assess quality of life in these populations; quality of life has been identified as a core outcome domain by the ongoing Outcome Measures for VAscular MAlformations (OVAMA) project [60–64].

## Current and emerging treatment options

Current treatment options include debulking surgery, sclerotherapy, and laser therapy [6, 35, 39, 65, 66]. However, recurrence after surgery is common, necessitating repeated surgical procedures [1, 35, 66], which may result in functional complications [66, 67]. Moreover, these modalities and medical interventions such as prophylactic use of antibiotics, anticoagulants, and pain management treat patients’ symptoms rather than the underlying molecular etiology of PI3K pathway hyperactivation.

### Current and emerging targeted agents

Systemic agents under investigation for *PIK3CA*-related disorders target different components of the PI3K signaling pathway (Fig. 1).

#### Sirolimus

Sirolimus inhibits mTOR, which is a node in the PI3K signaling pathway, and has been investigated in several clinical trials in patients with vascular malformations and overgrowth disorders [1, 6, 21, 53, 54]. Sirolimus is currently used in an off-label capacity to treat these disorders [36, 57].

Sirolimus has shown efficacy in multiple phase 2 studies in this setting. In a study of patients with complicated vascular anomalies, including tumors and malformations (N = 61), 85% of patients who completed 12 courses of treatment (n = 53) had a partial response [53]. In a subgroup of patients with complex lymphatic anomalies (N = 18), 83% of patients showed improvement after sirolimus treatment [59]. Sirolimus has also demonstrated efficacy in patients with complex slow-flow malformations (N = 19) [54]. In the multi-institutional phase 2 PROMISE study of 39 patients with a confirmed *PIK3CA* variant and progressive overgrowth (a different population from that of the study of complicated vascular anomalies), sirolimus showed modest efficacy at lower doses but only limited reduction in adverse events compared with higher doses [1, 68]. Results are awaited from the completed phase 2 PERFORMUS trial of sirolimus in pediatric patients with complicated superficial slow-flow vascular malformations [69, 70], and from the ongoing phase 3 VASE trial (EudraCT:2015-001703-32) [37, 71].

The safety profile of sirolimus in clinical trials has been encouraging; common grade 1–2 adverse events include headache, fatigue, cutaneous rash, mucositis, gastrointestinal troubles, and flu-like syndrome [21, 57]. A common grade 3 adverse event is mucositis [57]. Although severe opportunistic infections (e.g., *Pneumocystis*) have not been observed to date in the VASE study, patients at particular risk of these infections (i.e., very young children, those with dose-dependent lymphopenia or neutropenia, or other comorbidities) should be strongly considered for dose reduction and/or *Pneumocystis* prophylaxis [21]. A retrospective multicenter chart review of off-label use of sirolimus in patients with vascular anomalies (N = 113) identified 17 severe adverse events in 14 patients, most frequently viral pneumonia, highlighting the risk of severe and potentially fatal adverse events in these patients [72].

Several case reports/case series with sirolimus have also reported outcomes in this population [22, 68, 70, 73, 74]. Ongoing studies with sirolimus in *PIK3CA*-related disorders are described in Table 2. Primary endpoints in the ongoing studies include volumetric changes assessed by MRI or ultrasound, improvement in clinical lab indexes, adverse events, and patient-reported outcomes (NCT04598204, NCT02638389, NCT03767660).

**Table 2:** Select ongoing clinical trials of *PIK3CA*-related disorders using systemic therapies

Therapy	Study name (if applicable)	Clinical trial identifier	Phase or study type	Estimated number of patients	Patient population	Confirmed *PIK3CA* mutation required
Sirolimus	–	NCT04598204	2/3	30	1 month to 14 years old with Kaposiform Hemangioendotheliomas, Tufted Angioma, or complicated vascular malformation	No
	–	NCT02638389 EudraCT: 2015-001703-32	3	250	3 months to 70 years old with complex vascular anomalies	No
	–	NCT03767660	4	20	BRBNS, VMCM, sporadic multiple VM, or large single VM	No
Miransertib	–	NCT03317366	Expanded access	Not available	2 years and older with overgrowth diseases and/or vascular anomalies	Yes (*PIK3CA* or *AKT*)
	MOSAIC	NCT03094832	1/2	85	2 years and older with PROS or PS	Yes (*PIK3CA* or *AKT1*)
Alpelisib	–	NCT04085653	Managed access	Not available	2 years and older with PROS	Yes
	EPIK-P1	NCT04285723	Chart review	65	2 years and older with PROS	Yes
	EPIK-P2	NCT04589650	2	150	2 years and older with PROS	Yes

BRBNS: Blue Rubber Bleb Nevus Syndrome; PROS: *PIK3CA*-related Overgrowth Spectrum; PS: Proteus syndrome; VM: venous malformation; VMCM: venous malformation cutaneo-mucosal

#### Miransertib

Miransertib, an oral, allosteric inhibitor of AKT, demonstrated antiproliferative activity in primary fibroblasts derived from patients (N = 6) with *PIK3CA*-related disorders in the presence or absence of growth factors [75]. In preliminary results of an open-label, phase 1/2 study of miransertib in patients (N = 15) with *PIK3CA*-related Overgrowth Spectrum and Proteus Syndrome (MOSAIC), there was radiologically-confirmed lesion stability, and most patients experienced improvement in Karnofsky/Lansky performance scale, movement fluidity, fatigability, and pain relief (Table 2) [33, 56]. A case series with miransertib in two children with severe PROS reported an objective clinical response in a patient with CLOVES and reduced seizure frequency and improved quality of life in a patient with FIL and HMEG; no significant adverse events were reported [76].

#### Alpelisib

Alpelisib selectively inhibits PI3Kα and therefore directly targets the effects of *PIK3CA* activating mutations [55, 77]. In a study in human umbilical vein endothelial cells with *PIK3CA* or *TEK* mutations, alpelisib reversed abnormal AKT phosphorylation, cell morphology, and extracellular fibronectin levels—further highlighting the connection between TIE2 and PI3Kα in the etiology of common venous malformations. Notably, sirolimus did not restore extracellular fibronectin to wild-type levels in these cells, which suggests that PI3K activity functions upstream or parallel to the AKT/mTOR axis may also contribute to disease pathology [4].

Alpelisib also demonstrated efficacy in a mouse model of PROS/CLOVES, and a study of 19 patients with various PROS disorders revealed treatment with alpelisib led to substantial clinical improvement and a radiological response in all patients [55]. Alpelisib also achieved notable improvements in severe symptoms for individual patients that include opioid dependency and mobility (n = 2), chronic gastrointestinal bleeding (n = 3), and cognitive function (two patients with MCAP/M-CM) [55]. Case reports have described the successful use of alpelisib in patients with CLOVES and PROS [78, 79]. Ongoing alpelisib studies are described in Table 2. Primary endpoints in the ongoing studies include response as indicated by reduction in lesion volume assessed by imaging (NCT04285723, NCT04589650).

## Conclusion

There are critical unmet needs for clinicians who treat *PIK3CA*-related disorders as well as for patients and caregivers. Management of patients with *PIK3CA*-related disorders requires a multidisciplinary approach to address the spectrum of potential comorbidities [6, 29, 36, 80, 81]. Patients face challenges with continued care as they age into adulthood and have limited access to specialists with adequate expertise with these disorders and emerging systemic treatments [21, 37, 57].

Given the rarity of these disorders and the amount of expertise required, a limited number of centers can offer optimal care. In the US, comprehensive centers for vascular anomalies are often affiliated with children’s hospitals in major cities. The Consortium of iNvestigators of Vascular AnomalieS (CaNVAS; https://www.chop.edu/centers-programs/consortium-investigators-vascular-anomalies-canvas) is composed of 16 US institutions and was founded by pediatric hematologist/oncologists and patient advocacy groups. In the EU, multidisciplinary centers for overgrowth as well as vascular anomalies offer comprehensive care—some of these centers are members of the European Reference Network on Rare Multisystemic Vascular Diseases (VASCERN: https://vascern.eu/). VASCERN’s ongoing establishment of national networks enables follow-up closer to home and advances in virtual care options, such as the Clinical Patient Management System wherein physicians may ask for diagnostic and management assistance for any VASCERN working group disease. However, many patients with *PIK3CA*-related disorders and their families continue to rely on local physicians who lack experience treating these rare disorders.

New therapies are needed to provide an alternative or supplement to existing therapies that treat symptoms without targeting the underlying cause of these disorders. Inhibitors of the PI3K pathway are currently under clinical investigation and have shown promising results. However, efficacy and safety data from off-label use of these agents in the real-world setting are limited, and patients frequently have underlying comorbid conditions. Because these agents will likely be used in the pediatric setting, data from prospective clinical trials on their long-term safety and pharmacokinetic profiles in young patients are critical. Clinicians will also need to determine how to optimally integrate these emerging targeted therapies into current treatment strategies, and projects are ongoing to develop guidance for local use, which could reduce the frequency of adverse events.

## Supplementary Information

**Additional file 1.** Plain language summary.

## Data Availability

Not applicable.

## Abbreviations

CLOVES: Congenital lipomatous overgrowth, vascular malformations, epidermal nevi, scoliosis/skeletal and spinal
CLVM: Capillary lymphatic venous malformation
DCMO: Diffuse capillary malformation with overgrowth
DMEG: Dysplastic megalencephaly
FAO: Fibroadipose hyperplasia
FAVA: Fibroadipose vascular anomaly
FIL: Fibroadipose or facial infiltrating lipomatosis
GLA: Generalized lymphatic anomaly
HH: Hemihyperplasia
HHML: Hemihyperplasia-multiple lipomatosis
HMEG: Hemimegalencephaly
LON: Lipomatosis of nerve
LVM: Lymphatic venous malformation
MCAP: Megalencephaly-capillary malformation
PI3K: Phosphatidylinositol-3-kinase
*PIK3CA*: Phosphatidylinositol-4,5-bisphosphate 3-kinase catalytic subunit alpha
PIP_3_: Phosphatidylinositol-3,4,5-trisphosphate
PROS: *PIK3CA*-related overgrowth spectrum
PS: Proteus syndrome
VM: Venous malformation
VMCM: Venous malformation cutaneo-mucosal
