# Supplementary material for: A review of mechanisms of disease across PIK3CA-related disorders with vascular manifestations
Source: Orphanet J Rare Dis. 2021 Jul 8;16:306. doi: 10.1186/s13023-021-01929-8 (PMC8268514; doi:10.1186/s13023-021-01929-8)
Supplement: Supplementary file 1 — Additional file 1. Plain language summary. [file 13023_2021_1929_MOESM1_ESM.pdf]

Plain Language Summary of
"A Review of Mechanisms of Disease Across *PIK3CA*-Related Disorders With Vascular Manifestations"

Guillaume Canaud, Adrienne Hammill, Denise Adams, Miikka Vikkula, Kim M. Keppler-Noreuil

What are *PIK3CA*-related conditions (or disorders)?

The term "*PIK3CA*-related conditions" describes a group of rare and diverse conditions caused by ***PIK3CA*** **mutations**

PIK3CA-related conditions

- *PIK3CA*-Related Overgrowth Spectrum (PROS)
- *PIK3CA*-Related Vascular Malformations
- Other *PIK3CA*-Related Lesions

- CLOVES syndrome, K-T syndrome, MCAP/M-CM, and other conditions with abnormal growth fall under the umbrella term of "PROS"
  - Therefore, a person with CLOVES syndrome is also considered to have a *PIK3CA*-related condition
- However, not all people with these conditions experience overgrowth—some only have vascular malformations (differences in blood or lymph/lymphatic vessels) or other kinds of lesions (such as abnormal growth of the skin, or abnormalities in the brain such as FCD)

Why are some people more affected than others?

- Within each condition, there is a range of physical features and every person is uniquely affected
- Features also vary greatly between conditions

Some people may have differences in their blood vessels or their lymphatic system

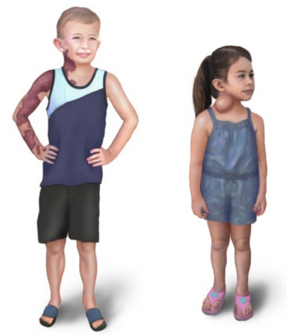

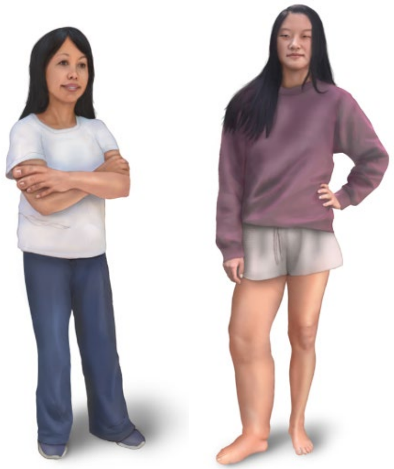

Some may have abnormal growth of one area of the body (known as focal overgrowth—shown here), whereas others may have abnormal growth in multiple areas (known as segmental overgrowth)

CLOVES, congenital lipomatous overgrowth, vascular malformations, epidermal nevi, scoliosis/skeletal and spinal; FCD, focal cortical dysplasia; K-T, Klippel-Trenaunay; MCAP/M-CM, megalencephaly-capillary malformation; *PIK3CA*, phosphatidylinositol-4,5-bisphosphate 3-kinase catalytic subunit alpha.

Why are some people more affected than others? (cont)

- *PIK3CA*-related conditions are caused by changes or mutations in a gene called *PIK3CA*
- The type of mutation (or **variant**) affects how severe the condition is
- In these conditions, *PIK3CA* mutations are **somatic**—meaning that they are new mutations (not inherited) and are only present in some cells or some areas of the body

The mutation first occurs sporadically in one cell during development of the embryo

Then the mutation is passed down to all cells that come from the abnormal cell

As the embryo develops, both normal and abnormal cells continue to multiply, forming a “**mosaic pattern**” that is unique and depends on where and when the mutation first appears

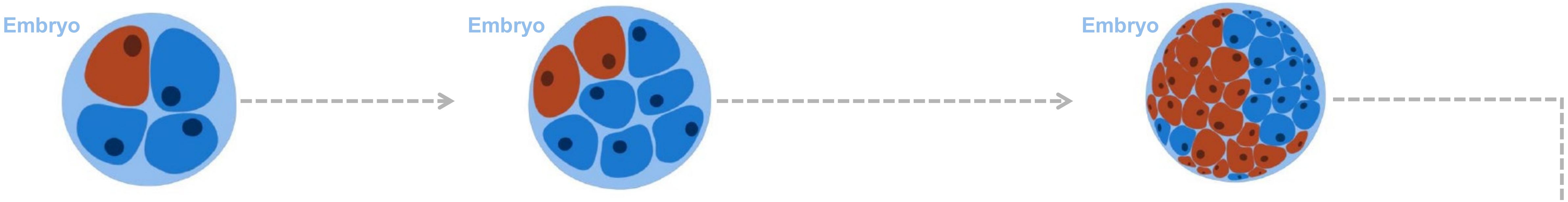

The *PIK3CA* variant and the timing and place of the mutation determine how severe or widespread the disease is

Widespread disease arises from early mutations, while isolated single lesions arise from later mutations

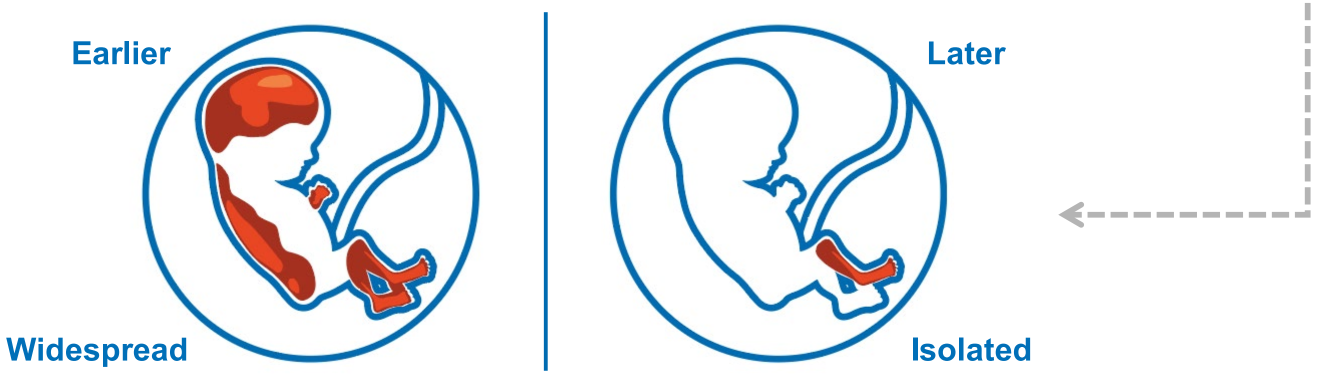

PIK3CA, phosphatidylinositol-4,5-bisphosphate 3-kinase catalytic subunit alpha.

What is the function of *PIK3CA*?

- The *PIK3CA* gene provides instructions to make a protein called **PI3K-alpha** (or PI3Kα)
- PI3K-alpha is part of the PI3K signaling pathway, which is involved in several functions including cell survival and cell division
- Mutations that occur in *PIK3CA*-related conditions result in increased PI3K-alpha activity leading to abnormal cell functions
- Several drugs that target proteins in this pathway are being investigated for use in treating *PIK3CA*-related conditions, including sirolimus (mTOR), miransertib (AKT), and alpelisib (PI3K-alpha)

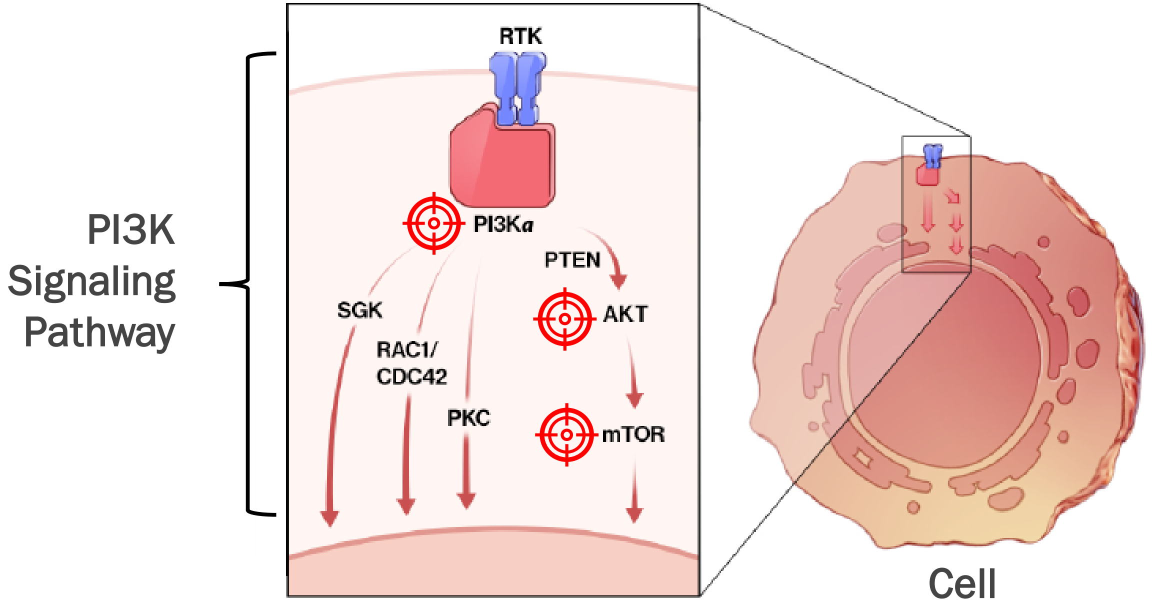

What kinds of complications are associated with *PIK3CA*-related disorders?

Depending on the location and severity of the condition, some of the following complications can occur:

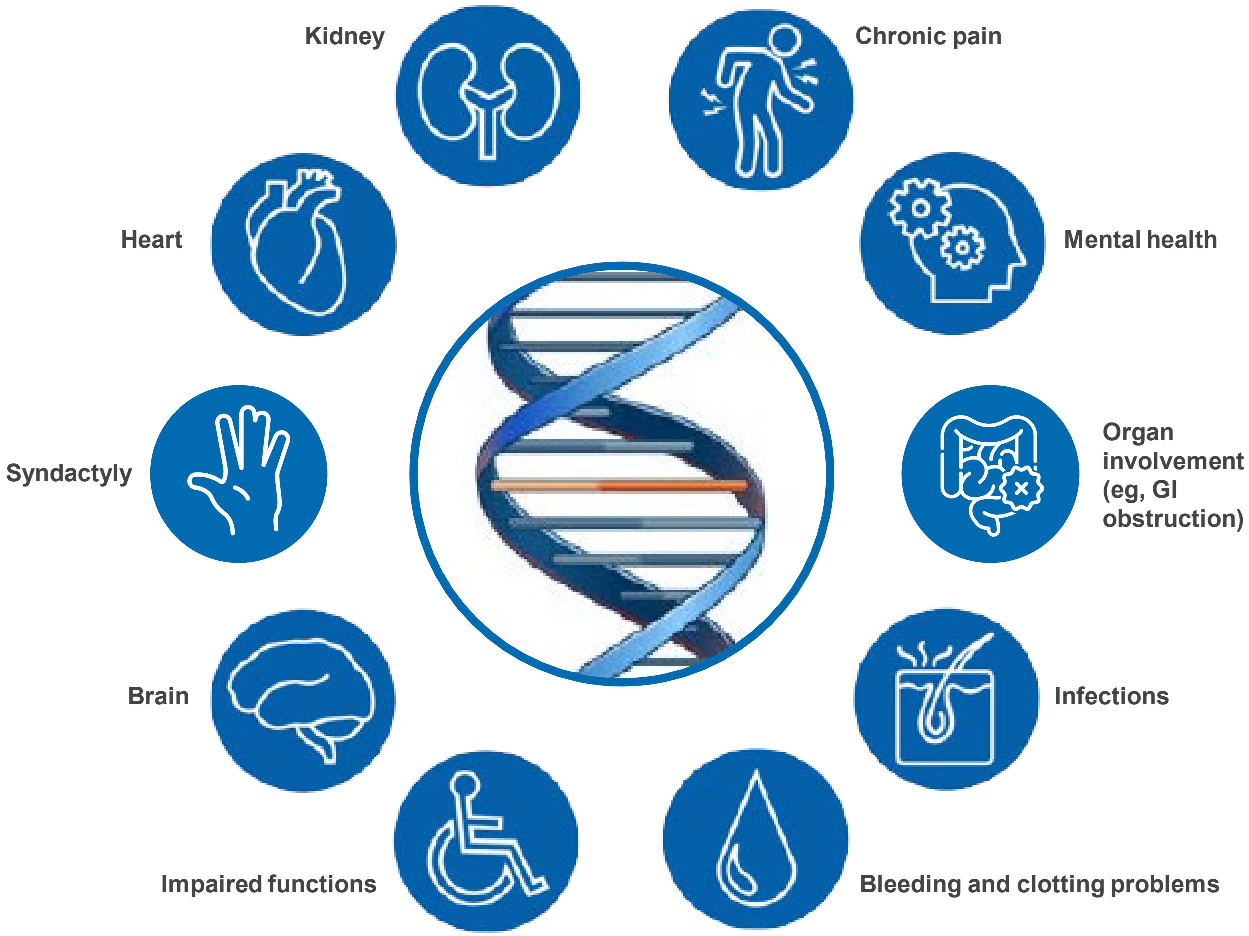

GI, gastrointestinal; PI3K, phosphatidylinositol-3-kinase; *PIK3CA*, phosphatidylinositol-4,5-bisphosphate 3-kinase catalytic subunit alpha.
